# Pharmacological Management of Thrombosis: Current State and Future Strategies

**DOI:** 10.3390/ijms27073151

**Published:** 2026-03-30

**Authors:** Andrzej Mogielnicki

**Affiliations:** Department of Pharmacodynamics, Medical University of Bialystok, 15-089 Bialystok, Poland; andrzej.mogielnicki@umb.edu.pl

**Keywords:** thrombosis, platelets, anticoagulant, antiplatelet, heparin, warfarin

## Abstract

Thromboembolic disorders remain a primary cause of mortality globally, with their burden expected to increase due to an aging population. While various antithrombotic agents exist, many patients still rely on traditional gold-standard drugs like heparin, warfarin, and aspirin, which present significant drawbacks. These include the need for injections, frequent monitoring, dietary restrictions, drug interactions, bleeding complications, or other adverse reactions such as thrombocytopenia. In contrast, direct oral anticoagulants offer advantages over warfarin, including fewer interactions and less need for continuous monitoring. However, even with newer drugs, patients face risks of major bleeding events. Current research focuses on novel antithrombotic agents with superior efficacy and enhanced safety compared to existing treatments. Researchers are exploring specific clinical niches, such as factor XI/XII inhibitors for cancer patients, to facilitate evaluation against standard treatments. The development of new antiplatelet drugs, like subcutaneous zalunfiban and selatogrel for pre-hospital therapy of myocardial infarction, and specific reversal agents, such as bentracimab for ticagrelor, exemplify this trend. Future research will likely focus on addressing the unmet needs of specific patient groups. This narrative review aimed to describe the current antithrombotic treatment and the most promising advances in the pharmacological management of thrombosis.

## 1. Thrombosis Development

Thrombotic diseases originate from disturbances in physiological hemostasis, a delicate balance that maintains blood flow while ensuring a rapid response to vascular injury. The maintenance of this equilibrium involves platelets and other blood cellular components, coagulation factors, the fibrinolytic system, and the vascular wall, particularly the vascular endothelium. The process is regulated through communication via activators and inhibitors of platelets and coagulation, as well as through additional mediators with pro- or antithrombotic properties. Pathological processes affecting any component of hemostasis can disrupt this balance, leading to a predisposition towards either thrombosis or bleeding [1].

**Figure 1:**
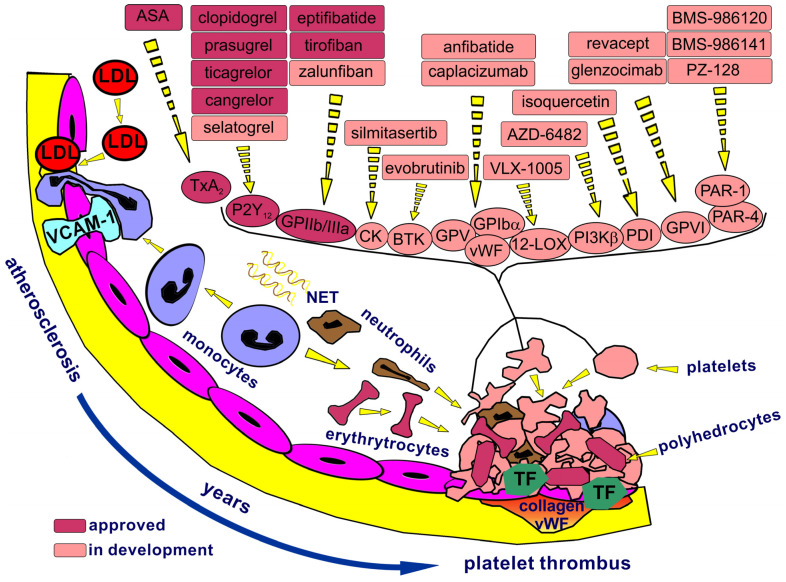
Atherothrombosis development and current and potential antiplatelet agents with their targets of action. TF—tissue factor; LDL—low-density lipoprotein; VCAM-1—vascular cell adhesion molecule 1; vWF—von Willebrand factor; Pharmacological targets: TxA_2_—thromboxane A_2_; P2Y_12_—adenosine diphosphate (ADP) receptor; GP—glycoprotein; PAR-1—proteinase-activated receptor 1; PAR-4—proteinase-activated receptor 4; PDI—protein disulfide isomerase; 12-LOX—12-lipoxygenase; PI3Kβ—phosphoinositide 3-kinase-β; BTK—tyrosine-protein kinase; CK—casein kinase; dashed arrows—inhibition.

One of the most frequent causes of thrombotic events is the dysfunction of the blood vessel wall and its inner lining, the vascular endothelium. This dysfunction frequently results from the chronic and progressive nature of the atherosclerotic process. Atherosclerosis, a progressive lipoprotein-driven disease, is characterized by the formation of atherosclerotic plaques at specific sites within the arterial vasculature (Figure 1). Atherosclerotic plaques may become unstable and prone to rupture, particularly at regions where the fibrous cap is thinnest and exhibits the most significant infiltration of foam cells. With plaque rupture, cap collagen and the highly thrombogenic lipid core, enriched in tissue factor (TF)-expressing apoptotic microparticles, activate both platelets and the coagulation system (Figure 1).

**Figure 2:**
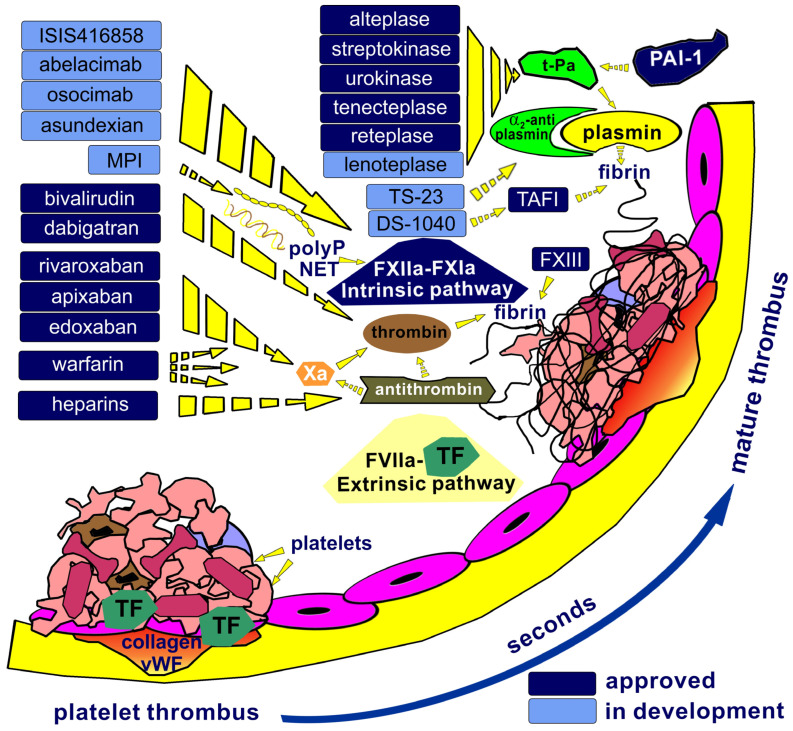
Thrombosis development and current and potential anticoagulants and thrombolytic agents with their mechanism of action. TF—tissue factor; FVIIa—Factor VIIa; Xa—factor Xa; FXIa—factor XIa; FXIIa—factor XIIa; NET—neutrophil extracellular traps; vWF—von Willebrand factor; t-PA—tissue plasminogen activator; PAI-1—plasminogen activator inhibitor-1; TAFI—thrombin-activatable fibrinolysis inhibitor; dashed arrows—inhibition.

Endothelial damage impairs its antithrombotic functions, including the release of prostacyclin and nitric oxide and the expression of ectonucleotidases that degrade platelet activators: adenosine triphosphate and diphosphate (ADP). Platelet adhesion to the injury site is primarily mediated by the glycoprotein (GP) GPIb-V–IX complex binding to subendothelial collagen via von Willebrand factor (vWF). Activated platelets release ADP and thromboxane A2, recruiting and activating more platelets. Thrombin further amplifies platelet activation and links platelet and coagulation pathways. Activated platelets express GPIIb/IIIa (integrin αIIbβ3), which facilitates platelet aggregation through the binding of fibrinogen. Exposed TF binds factor VII (FVII), initiating the extrinsic pathway of coagulation (Figure 2).

Amplification of thrombin generation requires the intrinsic pathway (FVIII, FIX, FXI, and FXII) due to the rapid inactivation of the TF-FVIIa complex by tissue factor pathway inhibitor. Given sufficient thrombin generation, the cofactors FV and FVIII are activated, fibrinogen is converted to fibrin, and active FXIII stabilizes the fibrin clot (Figure 2). Furthermore, thrombin and other agonists induce platelet activation, which provides a negatively charged phospholipid surface that substantially amplifies thrombin generation through the assembly of the intrinsic tenase (FVIIIa–FIXa) and prothrombinase (FVa–FXa) enzyme complexes [1].

The fibrinolysis process limits the propagation of thrombus to the site of vascular injury. The principal enzyme, plasmin, is responsible for the degradation of fibrin. Plasmin activity is locally regulated by circulating α2-antiplasmin (Figure 2). Furthermore, the natural fibrinolytic process is regulated by the activity of tissue plasminogen activator (t-PA) and its inhibitor, plasminogen activator inhibitor-1 (PAI-1), and typically progresses over days or even weeks until thrombus lysis and vessel recanalization occur, if achievable [2].

Beyond the aforementioned blood elements, erythrocytes play mainly a mechanical role in the development of thrombosis by influencing hemorheology. They generate thrombin through the exposure of phosphatidylserine and form polyhedrocytes, creating barriers that stabilize thrombi and reduce fibrinolysis, thereby promoting thrombus formation and persistence [3,4]. They also actively contribute to thrombosis by binding to platelets via the ICAM4-αIIbβ3 interaction, enhancing platelet activation, thrombin formation, and fibrin deposition [5].

The acceleration of the coagulation cascade involves not only negatively charged phospholipids of platelet membranes but also other negatively charged polyanionic structures, including polyphosphates (polyP) released from platelet granules and neutrophil extracellular traps (NET) composed of DNA and histone fragments [6] (Figure 2). These structures provide critical surfaces for the activation of key coagulation factors [7]. Impaired blood flow supports the formation of a stable, mature fibrin-crosslinked thrombus, composed of red blood cells, platelets, and leukocytes, that occludes the vessel lumen, leading to ischemia and serious cardiovascular complications. The localization of a thrombus influences clinical manifestations and the risk of severe complications, including myocardial infarction and ischemic stroke resulting from arterial thrombosis, deep vein thrombosis, and pulmonary embolism (PE) as consequences of venous thrombosis. Given an aging global population, the prevalence of these diseases is anticipated to rise, thereby underscoring the necessity for effective and, crucially, safe anticoagulant therapies for elderly individuals.

## 2. Current State of Pharmacological Thrombosis Management

Based on their mechanism of action, antithrombotic drugs can be classified into agents that inhibit platelet activity (antiplatelet drugs), agents that inhibit coagulation factors (anticoagulants), and agents that activate fibrinolysis (thrombolytic or fibrinolytic agents). Despite the passage of time, foundational drugs such as aspirin, heparin, and warfarin remain cornerstones of antithrombotic therapy. Furthermore, in specific clinical indications—for instance, warfarin for prophylaxis following prosthetic heart valve implantation and aspirin for long-term secondary cardiovascular prevention—these traditional agents remain the exclusive therapeutic choices.

### 2.1. Anticoagulants

**Heparin**, a glycosaminoglycan, was initially isolated from canine liver [8] and subsequently sourced from bovine lungs and porcine intestinal mucosa [9]. It consists of sulfated chains of uronic acid and glucosamine and has a molecular weight range of 2–40 kDa [10]. It exerts its anticoagulant effect by potentiating antithrombin, an endogenous coagulation inhibitor, thereby inhibiting the activity of factor Xa (fXa) and thrombin to a similar extent. Unfractionated heparin (UFH), as it is called, is administered intravenously to patients at risk of or experiencing myocardial infarction or ischemic stroke, and it is used for the prophylaxis and treatment of venous thromboembolism (VTE) and PE. With a rapid action (half-life around 1.5 h), it also constitutes a fundamental component of parenteral perioperative anticoagulation strategies (Table 1).

**Low-molecular-weight heparins (LMWHs)**, derived from UFH via chemical or enzymatic depolymerization, possess shorter polysaccharide chains and are suitable for subcutaneous administration. The biological activity of UFH and related LMWHs is determined primarily by charge distribution, sulfation pattern, chain length, and conformational flexibility. QSAR analyses consistently identify the highly sulfated pentasaccharide sequence as the essential pharmacophore responsible for high-affinity antithrombin binding. The mechanism of action of LMWHs differs from that of UFH, exhibiting greater selective inhibition of fXa relative to thrombin. They demonstrate a more predictable anticoagulant response, permitting administration via fixed or weight-adjusted doses without requiring routine coagulation monitoring. The half-lives of LMWHs vary: dalteparin has a mean retention time of approximately 5 h, while enoxaparin and nadroparin have approximately 7 h [11] (Table 1). Their indications are similar to UFH; however, their suitability for once-daily self-administration due to a longer action allows for chronic outpatient use in the prevention and treatment of thromboembolic disorders, both as monotherapy and in combination with other drugs. Additionally, they are associated with a lower incidence of heparin-induced thrombocytopenia (HIT), a potentially life-threatening adverse drug reaction associated with a high risk of thrombosis [12].

The main absolute contraindications for heparin use include active bleeding, hemorrhagic diathesis, and severe thrombocytopenia. Other key ones are recent hemorrhagic stroke, head trauma or surgery, uncontrolled hypertension, severe renal or liver failure, and peptic ulcer disease. Heparin is also avoided in aortic aneurysm, bacterial endocarditis, and ulcerative colitis.

The limitations and adverse effects of traditional standard antithrombotic agents prompted the development of novel anticoagulants in the early 2000s. In the case of heparins, chemically distinct agents were developed for parenteral use in acute settings, similarly to conventional heparins. A common characteristic of these novel drugs is their significantly lower molecular weight than heparins (Table 1).

**Fondaparinux** is the most structurally similar to heparins. It is a synthetic analog of the pentasaccharide sequence found in heparins approved by the FDA in 2001 [13]. Fondaparinux selectively binds to antithrombin, with minimal interaction with other plasma proteins, resulting in the selective inhibition of fXa. Its favorable pharmacokinetic properties include high subcutaneous bioavailability, a prolonged half-life, and consistent anticoagulant effects, enabling its administration without continuous laboratory monitoring (Table 1). Clinically, fondaparinux is indicated for the treatment of acute coronary syndrome, deep vein thrombosis, and PE. It is also approved for postoperative thromboprophylaxis [14]. Fondaparinux may replace heparin in clinically stable patients at average risk of bleeding in whom HIT occurred or is suspected [15]. Other agents that could be used as alternatives to heparin include **lepirudin and bivalirudin**, which were approved by the FDA in 1998 and 2000, respectively. These are analogs of hirudin, a natural thrombin inhibitor derived from the saliva of the medicinal leech. In contrast, **argatroban**—another heparin alternative—is a synthetic compound derived from L-arginine, a molecule with intrinsically weak antithrombotic activity [16]. Lepirudin, a recombinant protein produced in yeast cells, was withdrawn from the market in 2012 due to high production costs and a significant risk of immunogenic reactions. Unlike heparins, bivalirudin and argatroban directly, reversibly, and selectively inhibit thrombin. Both thrombin inhibitors share similar narrow contraindication profiles focused on active major bleeding and hypersensitivity to the drug. Bivalirudin is mainly eliminated via the renal route, whereas argatroban undergoes hepatic metabolism. Thus, bivalirudin requires dose adjustment (not contraindicated) in renal impairment, while argatroban demands caution in hepatic dysfunction, with activated partial thromboplastin time (aPTT) monitoring essential for both. In patients with critical conditions, an increased risk of bleeding, and a potential need for urgent procedures, argatroban or bivalirudin are preferred as heparin replacements over fondaparinux due to their shorter duration of action [15].

While these newer anticoagulants offer specific advantages, they have not universally replaced heparin. UFH effects can be readily reversed with protamine sulfate, a crucial advantage in cases of overdose or the need for urgent procedures. Neither fondaparinux, bivalirudin, nor argatroban has a specific, widely available antidote, although bivalirudin’s short half-life allows for rapid offset of its effects upon discontinuation. Despite their improved pharmacodynamics and pharmacokinetics, the inability to be administered orally remains the primary limitation of all parenteral anticoagulants, particularly for long-term prophylaxis. In chronic oral anticoagulant therapy, **warfarin** remains the traditional gold standard. It is a derivative of natural compounds found in plants rich in coumarins. Warfarin acts by inhibiting the vitamin K epoxide reductase complex subunit 1 (VKORC1), thereby impairing the activation of vitamin K-dependent clotting factors II, VII, IX, and X, as well as the anticoagulant proteins C and S. QSAR analysis of warfarin demonstrates that anticoagulant activity arises from an interplay of hydrophobicity, electronic distribution, steric geometry, and stereochemistry centered on the 4-hydroxycoumarin scaffold. Both classical and 3D-QSAR models consistently highlight the importance of phenyl side-chain orientation and optimal lipophilicity for VKORC1 inhibition. It is typically administered once daily, with dosing highly individualized due to variability in metabolic rate, dietary vitamin K intake, comorbid conditions, and potential drug interactions. Because warfarin produces only a partial anticoagulant effect after approximately 2–3 days, with full antithrombotic activity typically requiring about 5 days, initial overlap with rapidly acting heparin is required during the early phase of acute VTE treatment [17,18]. Monitoring of regular international normalized ratio (INR), a validated prothrombin time (PT), is essential, with therapeutic targets generally ranging from 2.0 to 3.5, depending on the clinical indication. Warfarin remains a widely used oral anticoagulant for both the prevention and treatment of thromboembolic disorders. FDA-approved indications include prophylaxis and treatment of VTE, thromboembolic events associated with AF and prosthetic heart valves, and reduction in thrombotic risk post-myocardial infarction. The strongest clinical justification for preferring warfarin over direct oral anticoagulants (DOACs) lies in patients with mechanical prosthetic heart valves, antiphospholipid syndrome, or those at high risk of gastrointestinal bleeding, for whom DOACs have not demonstrated equivalent safety and efficacy [19]. The 4-hydroxyl group of warfarin contributes to high protein binding (>99% albumin binding). It is associated with numerous drug–drug interactions, particularly with nonsteroidal anti-inflammatory drugs, antibiotics, and other anticoagulants and antiplatelet agents, necessitating close monitoring during co-administration. Besides drug interactions, interindividual variability in warfarin response is influenced by comorbidities and genetic polymorphisms affecting its metabolism and pharmacodynamics. Approximately 30–40% of the variability in dose requirements is attributable to genetic variants, particularly within the *CYP2C9* and *VKORC1* genes. Allelic variants, such as CYP2C9*2 and CYP2C9*3, lead to reduced enzymatic activity and slower warfarin clearance, thereby increasing the risk of bleeding at standard doses [20]. Furthermore, the *VKORC1*-1639G>A polymorphism results in heightened warfarin sensitivity due to decreased expression of the enzyme target, thereby necessitating lower therapeutic doses of warfarin. In addition to *VKORC1*-1639G>A, other linked polymorphisms—particularly rs9934438 (1173C>T)—are associated with increased warfarin sensitivity and reduced maintenance dose requirements. These variants largely tag the low-dose *VKORC1* haplotype group A, which is characterized by lower *VKORC1* expression and enhanced pharmacodynamic response to warfarin [21,22,23]. Although the influence of pharmacogenetic variability on warfarin response is well documented [20], clinical guidelines do not recommend routine genotyping before treatment initiation [24]. Warfarin is contraindicated in active clinically significant bleeding, hypersensitivity, pregnancy, severe liver disease with coagulopathy, and hemorrhagic diathesis. Additional absolute contraindications include recent intracranial hemorrhage, recent neurosurgery or ocular surgery, peptic ulcers, frequent falls, alcoholism, psychosis, or dementia impairing compliance. The narrow therapeutic window and challenges associated with dosing and maintaining stable anticoagulation with warfarin have driven the development of DOACs.

**Dabigatran**, a direct thrombin inhibitor derived from melagatran and ximelagatran, was the first DOAC approved by the FDA in 2010 [25]. Pharmacodynamically, dabigatran is a reversible, competitive thrombin inhibitor that prevents the conversion of fibrinogen to fibrin. Due to this mechanism of action, a close correlation exists between the drug’s blood concentration and its anticoagulant effect [26]. It is administered orally as dabigatran etexilate, a prodrug converted to the active form by serum esterases (Table 1). Its oral bioavailability is approximately 7%, enhanced by co-formulation with tartaric acid, which facilitates absorption in an acidic gastric environment and may contribute to gastrointestinal side effects. Dabigatran is primarily renally excreted as the “unchanged form” (~80%); thus, dose reduction is required in renal impairment [27].

The approved oral factor Xa inhibitors—**rivaroxaban**, **apixaban**, and **edoxaban**—selectively and reversibly inhibit activated factor X, thus preventing thrombin generation. QSAR analyses of dabigatran, rivaroxaban, apixaban, and edoxaban indicate that anticoagulant activity is governed by a shared set of key structural determinants: electrostatic complementarity to the target enzyme, optimized hydrogen-bonding patterns, controlled molecular polarity, and steric adaptation to protease binding pockets. Dabigatran activity depends primarily on strong ionic and hydrogen-bond interactions within thrombin’s catalytic site, whereas factor Xa inhibitors rely more heavily on aromatic stacking and hydrophobic occupancy of S1/S4 subsites. Across all DOACs, QSAR models highlight the importance of balancing polarity with lipophilicity to achieve oral bioavailability while maintaining high target selectivity.

Renal clearance differs across Xa inhibitors (edoxaban ~50%, rivaroxaban ~35%, and apixaban ~25%), and all are substrates for P-glycoprotein. Rivaroxaban and apixaban are further metabolized via the CYP3A4 pathway, unlike edoxaban. Notably, rivaroxaban absorption increases by approximately 40% when taken with a high-fat meal, necessitating administration with food [28].

The elimination half-lives of DOACs do not consistently correspond to their dosing regimens. Dabigatran has a half-life of approximately 7–9 h after a single dose and 14–17 h after multiple doses, yet it is administered twice daily, as is apixaban, despite its half-life of approximately 12 h. Conversely, rivaroxaban is given once daily, although its half-life is only about 7 h. Of the currently available DOACs, only edoxaban, with a half-life of approximately 12 h, appears to align with once-daily administration (Table 1).

A series of pivotal randomized controlled trials comparing DOACs to warfarin in patients with AF demonstrated superior or comparable efficacy and improved safety [29]. Generally, the risk of major bleeding, including intracerebral hemorrhage and gastrointestinal bleeding, was significantly reduced with DOACs, in comparison to warfarin, with some exceptions. Specifically, major gastrointestinal bleeding was increased with higher doses of dabigatran and edoxaban and a regular dose of rivaroxaban [30]. A network meta-analysis of other RCTs reflected the results of pivotal trials on reduced intracerebral hemorrhage with all DOACs compared to warfarin and varied risk of major and gastrointestinal bleedings depending on the type of DOAC [31]. Thus, major bleeding, predictable pharmacology, and simplified perioperative management are key advantages of DOACs compared with warfarin. Still, DOACs share key absolute contraindications, such as active clinically significant bleeding, hypersensitivity, severe renal impairment, and hepatic impairment associated with coagulopathy or Child-Pugh C. Additional critical ones include pregnancy/breastfeeding, lesions/conditions with high bleeding risk (e.g., recent intracranial hemorrhage, spinal surgery, bacterial endocarditis), and concomitant treatment with other anticoagulants except in specific switching or catheter-related circumstances. While DOACs have become the preferred anticoagulant in many indications, warfarin still holds a significant place in therapy due to its lower cost, established use in specific patient populations, and the extensive clinical experience with its management.

### 2.2. Antiplatelet Drugs

Currently available antiplatelet drugs represent three main mechanisms of platelet inhibition depicted in Figure 1.

**Acetylsalicylic acid** (ASA) irreversibly acetylates cyclooxygenase-1, an enzyme used by platelets to generate the second messenger thromboxane A2, a potent platelet agonist [1] (Figure 1). At higher doses, ASA also inhibits cyclooxygenase-2, thereby preventing the synthesis of prostaglandin H2 [32]. A daily dose of 75–100 mg of ASA is sufficient to completely inhibit thromboxane A2 synthesis. QSAR analysis of acetylsalicylic acid highlights the critical balance among lipophilicity, electronic distribution, and hydrogen bonding that underlies its pharmacological activity. ASA is used in the pharmacotherapy of recent or suspected myocardial infarction, unstable angina, previous coronary angioplasty, and atherosclerosis of the lower limb arteries. The most favorable indication for aspirin use is in secondary prevention, where the risk of thrombosis is higher [33]. Low doses of ASA are recommended in long-term prevention. The CURRENT-OASIS 7 study confirmed that such doses provide comparable anti-ischemic efficacy while associated with fewer adverse events [34]. The role of high-dose aspirin (>300 mg) is largely confined to clinical settings requiring immediate, robust platelet inhibition, such as aspirin loading before or during PCI in acute coronary syndrome (ACS) and initiation of antiplatelet therapy after minor ischemic stroke or high-risk transient ischemic attack. Because chronic use of higher doses has not shown a meaningful efficacy advantage over low-dose aspirin but is associated with broader prostaglandin suppression and a higher risk of gastrointestinal toxicity, current practice generally limits such dosing to acute loading strategies or selected cerebrovascular indications, including severe symptomatic intracranial atherosclerotic stenosis, where aspirin 325 mg daily remains an accepted option [35,36]. ASA, in combination with an oral P2Y12 receptor inhibitor, constitutes dual antiplatelet therapy (DAPT), which is the cornerstone of antithrombotic treatment in patients undergoing elective PCI. The standard duration of DAPT is set at 12 months, primarily to prevent recurrent thrombotic events. While extending DAPT beyond this period offers protection against myocardial infarction and stroke, it is associated with an increased risk of bleeding; the mortality and incidence of intracerebral hemorrhage remain unaffected [37]. In maintenance therapy for patients with stable coronary artery disease who have undergone PCI, lifelong ASA administration alone is recommended. ASA is contraindicated in patients with hypersensitivity to salicylates or other non-steroidal anti-inflammatory drugs, active peptic ulcer disease or bleeding, hemorrhagic disorders, severe hepatic or renal impairment, gout, concomitant methotrexate therapy at doses >15 mg/week, and during the third trimester of pregnancy at doses >100 mg/day. Additional key restrictions apply to children under 16 (due to Reye’s syndrome risk).

The second group of antiplatelet drugs to be discovered after ASA are antagonists of purinergic receptors for ADP, the earliest of which was ticlopidine. ADP is a key platelet agonist, exerting its effects through binding to purinergic receptors P2Y1 and P2Y12. Activation of these receptors influences platelet aggregation. Stimulation of the P2Y1 receptor results in calcium influx, platelet shape change, and rapid but reversible aggregation. In contrast, activation of the P2Y12 receptor leads to the inhibition of adenylyl cyclase and a reduction in intracellular cyclic adenosine monophosphate levels. The decrease in adenosine monophosphate inhibits phosphorylation of vasodilator-stimulated phosphoprotein, which subsequently induces activation of the GPIIb/IIIa receptor and platelet aggregation [38]. Ex vivo aggregation studies have shown that all other platelet agonists depend to some extent on released ADP to elicit maximal platelet aggregation, although this dependence varies by agonist.

**Ticlopidine** was first introduced in the 1990s for the prevention of stroke and stent thrombosis [1]. However, ticlopidine use has been associated with serious hematological toxicities, particularly neutropenia/agranulocytosis, thrombotic thrombocytopenic purpura, and aplastic anemia [39]. This also led to the development of the second-generation P2Y12 antagonist, clopidogrel.

**Clopidogrel** is orally administered as a prodrug (Table 1). Following intestinal absorption, approximately 85% of clopidogrel is hydrolyzed by esterases into an inactive form, with only about 15% being metabolized into its active form via the cytochrome P450 (CYP450) [40]. Its active metabolite irreversibly binds to the P2Y12 receptor by forming a disulfide bridge between its reactive thiol group and a cysteine residue on the receptor. This binding prevents ADP from interacting with the receptor, inhibiting platelet adhesion and aggregation [41]. The CURE trial demonstrated the beneficial effects of clopidogrel in patients with non-ST-segment elevation acute coronary syndromes. However, a higher risk of major bleeding was observed in the clopidogrel-treated group [42]. The COMMIT trial included patients with acute myocardial infarction confirmed by ECG abnormalities. Clopidogrel reduced the relative risk of death from any cause by 7%, and the combined relative risk of recurrent myocardial infarction, stroke, or death by 9%. These benefits were observed regardless of age, sex, or the use of fibrinolytic therapy [43]. In combination with ASA as part of DAPT, it is recommended for patients with AF who cannot receive warfarin and have a low risk of bleeding. In ACS, clopidogrel is recommended only when other oral P2Y12 antagonists are unavailable or contraindicated. It may be considered in elderly patients exceeding 70 years of age [35]. Clopidogrel contraindications include hypersensitivity, severe hepatic impairment, active pathological bleeding (e.g., peptic ulcer or intracranial hemorrhage), recent severe bleeding from trauma/surgery, concomitant use with strong CYP2C19 inhibitors (e.g., omeprazole), and caution in moderate liver impairment or bleeding diathesis due to antiplatelet effects.

Patient responses to clopidogrel may vary due to polymorphisms in cytochrome P450, resulting in inadequate clopidogrel metabolism and suboptimal antiplatelet effects. This phenomenon is associated with genetic variants of the *CYP2C19* gene [44]. Resistance to clopidogrel prompted the development of third-generation thienopyridines, prasugrel, ticagrelor, and cangrelor. Like clopidogrel, newer oral P2Y12 receptor antagonists are indicated for preventing and inhibiting arterial thrombosis, most notably as part of DAPT in combination with ASA for patients with coronary artery disease [45].

**Prasugrel**, approved by the FDA in 2009, undergoes fewer hepatic metabolic transformations than clopidogrel, resulting in higher levels of its active metabolite. As a result, antiplatelet potency increases, and interindividual variability in drug response is reduced. It also has a more rapid onset of action, achieving peak platelet inhibition within 1 to 2 h following a 60 mg oral loading dose, with a mean half-life of 7 h (Table 1). Clinical evidence indicates that prasugrel is better at lowering the incidence of major adverse cardiovascular events in patients with acute coronary syndrome undergoing PCI [46]. Prasugrel is contraindicated in active pathological bleeding (e.g., peptic ulcer, intracranial hemorrhage), hypersensitivity, and severe hepatic impairment (Child-Pugh C). It is also contraindicated in patients with a history of stroke or transient ischemic attack because this subgroup has an unfavorable benefit-risk profile, primarily due to an increased risk of major, especially intracranial, bleeding [47,48].

**Ticagrelor** is an orally administered direct-acting and reversibly binding P2Y12 receptor antagonist approved by the FDA in 2011. It is not required to be activated via hepatic metabolism. It has a rapid onset of action, with a median time to maximum concentration ranging from 1.3 to 2 h after administration, and a mean half-life time of 6.3 h (Table 1) [49]. When compared to clopidogrel, it exhibits superior antiplatelet efficacy in patients with acute coronary syndrome or ischemic stroke, leading to a greater reduction in major adverse cardiovascular events and all-cause mortality, though accompanied by a higher risk of bleeding. Ticagrelor is contraindicated in patients with active pathological bleeding, a history of intracranial hemorrhage, or severe hepatic impairment. Beyond P2Y12-dependent mechanisms, ticagrelor exhibits additional pharmacodynamic effects, including anti-inflammatory properties and the induction of beneficial platelet-neutrophil interactions [46]. Ticagrelor also inhibits cellular adenosine reuptake via equilibrative nucleoside transporter 1, thereby increasing extracellular adenosine availability. This off-target effect may contribute to some of its pleiotropic vascular and cardioprotective effects, but it has also been implicated in the development of adverse effects such as dyspnea [50,51].

**Cangrelor** is a direct, reversible, intravenously administered P2Y12 receptor antagonist approved by the FDA in 2015. It does not require metabolic activation and has a plasma half-life of 2.9–5.5 min (Table 1) [52]. Clinical studies have demonstrated its efficacy in reducing short-term recurrent myocardial infarction compared to clopidogrel in patients undergoing PCI [46]. A meta-analysis of 15 RCTs with 54,025 patients undergoing PCI showed comparable clinical outcomes in groups treated with cangrelor, ticagrelor, or prasugrel [53]. The intravenous route and metabolism-independent action of cangrelor render it particularly useful in acute scenarios, for example, cardiogenic shock, or in patients unable to take drugs orally. Additionally, its favorable pharmacodynamic characteristics make cangrelor a promising candidate in situations requiring temporary discontinuation of oral P2Y12 inhibitors [46]. Cangrelor is contraindicated in patients with active bleeding or an increased risk of bleeding and in those with hypersensitivity to cangrelor or any of its excipients.

QSAR analysis of P2Y12 receptor antagonists highlights the critical role of molecular scaffolds and physicochemical properties in modulating antiplatelet activity. Clopidogrel and prasugrel, as thienopyridine prodrugs, rely on ester functionality and electronic/steric features that govern hepatic bioactivation and P2Y12 binding, with lipophilicity, polar surface area, and hydrogen bonding capacity correlating with potency and selectivity. In contrast, ticagrelor’s cyclopentyl-triazolo-pyrimidine core enables direct, reversible inhibition, where moderate lipophilicity and receptor-oriented steric features determine rapid onset and oral bioavailability. Cangrelor, a highly polar ATP analog, exhibits strong, fast-acting reversible antagonism in plasma, with its polarity and hydrogen bonding defining a short half-life and minimized off-target effects.

**Abciximab**, a chimeric mouse/human antibody, was the first GPIIb/IIIa receptor inhibitor approved by the FDA in 1994 [54]. Upon platelet stimulation, the GPIIb/IIIa receptor undergoes activation, transitioning to a high-affinity conformation that mediates platelet aggregation through binding to fibrinogen and other ligands. Abciximab is a potent competitor, and its complexes with platelets can persist for up to a week following administration, despite the rapid elimination of free plasma abciximab from circulation within minutes. The GPIIb/IIIa receptor inhibitors are a chemically diverse group of drugs. **Eptifibatide**, a cyclic heptapeptide, relies on conformational rigidity and key amino acid residues to optimize receptor binding. **Tirofiban**, a non-peptide small-molecule tyrosine derivative, achieves reversible inhibition through a constrained aromatic-acyl scaffold. They have smaller molecular weights and shorter half-lives than abciximab (Table 1). Differences also exist in their mechanisms of action. Abciximab binds non-specifically to integrin αIIbβ3 and other glycoproteins, such as αVβ3 [55]. Both newer drugs bind to the aspartic acid in the αIIb domain, but tirofiban, with multiple ligands, also binds to β3 [56]. While GPIIb/IIIa inhibitors have demonstrated efficacy in reducing thrombus burden and mitigating the risk of periprocedural thrombosis during PCI, their use was often associated with an elevated incidence of bleeding events and thrombocytopenia in patients with acute coronary syndrome. These factors likely contributed to the discontinuation of abciximab production in 2020 [39]. In ACS management, glycoprotein IIb/IIIa inhibitors are not used routinely. Current guidelines restrict their use to selected patients undergoing PCI, mainly as adjunctive bailout therapy in the presence of no-reflow, slow flow, large thrombus burden, or other thrombotic complications, whereas routine or upstream administration is discouraged because it has not shown clear ischemic benefit and increases bleeding risk [35,57].

Eptifibatide and tirofiban are contraindicated in patients with active or recent clinically relevant bleeding, prior hemorrhagic stroke or intracranial pathology, thrombocytopenia or coagulation disorders, severe uncontrolled hypertension, recent major trauma or surgery, severe hepatic impairment, and hypersensitivity.

### 2.3. Thrombolytic (Fibrinolytic) Agents

The first-generation fibrinolytic agents, **streptokinase** and **urokinase**, demonstrated mortality benefits in early trials for myocardial infarction. However, streptokinase’s lack of fibrin specificity led to systemic plasminogen activation and increased bleeding risk. It also induced immune and allergic reactions due to its origin, limiting repeated use. Streptokinase is contraindicated in patients with active or recent internal bleeding, recent cerebrovascular events, recent intracranial or intraspinal surgery, intracranial neoplasm, severe uncontrolled hypertension, major coagulation disorders, recent head trauma, hemorrhage-prone neoplasms, acute pancreatitis, and severe hypersensitivity. The contraindication in stroke is primarily related to the high risk of intracranial hemorrhage and the unfavorable benefit-risk profile reported in acute ischemic stroke trials [58,59,60]. Despite these drawbacks, streptokinase and urokinase remain in use in some developing countries due to their affordability and availability [61].

**Alteplase**, a recombinant t-PA, is a glycoprotein enzyme that, similarly to endogenous t-PA, promotes fibrinolysis by converting fibrin-bound plasminogen to plasmin, the primary enzyme responsible for degrading fibrin in thrombi [62]. It has a very short half-life of 3.5 min after a single intravenous bolus and is cleared rapidly primarily by the liver (Table 1) [63]. Alteplase is considered the “gold standard” fibrinolytic agent for the treatment of ischemic stroke, acute myocardial infarction, and PE [61]. However, alteplase is associated with increased risk of intracerebral hemorrhage and systemic bleeding. Its fibrin specificity, while superior to streptokinase, remains imperfect, and activation of circulating plasminogen occurs to a considerable degree. Consequently, systemic fibrinogen depletion ensues, leading to a prothrombotic state if thrombolysis is incomplete. In acute ischemic stroke, alteplase administration within 4.5 h of symptom onset improves functional outcomes [63]. Alteplase is contraindicated in patients with active bleeding, a known hemorrhagic diathesis or recent major bleeding-risk conditions; any history of hemorrhagic stroke in those with recent head trauma, severe uncontrolled hypertension, thrombocytopenia, marked coagulation abnormalities, or blood glucose <50 or >400 mg/dL.

These limitations have prompted the search for newer, more favorable mutation variants, including reteplase and tenecteplase [61]. A significant difference between alteplase and these agents is their longer half-lives and greater resistance to PAI-1 (Table 1) [61,64].

**Reteplase**, a nonglycosylated peptide with reduced fibrin affinity compared to alteplase, was created to improve plasma clearance and allow bolus administration. Reteplase has a half-life of 12.6 min and demonstrates improved coronary artery patency compared to alteplase in acute myocardial infarction. However, increased intracranial hemorrhage risk compared to alteplase has been observed [64].

**Tenecteplase**, a derivative of alteplase incorporating three point mutations in its kringle 1 and protease domains, exhibits greater fibrin specificity, reduced hepatic elimination, increased resistance to PAI-1, resulting in a decreased plasma clearance rate, and a prolonged plasma half-life of 22 min (Table 1) [65]. A recent meta-analysis suggested that tenecteplase has greater efficacy and a favorable safety profile for treating acute ischemic stroke within an extended time window [66]. It was approved by the FDA in 2025 for the treatment of acute ischemic stroke in adults [67].

According to the 2026 AHA/ASA guideline, alteplase and tenecteplase (0.25 mg/kg; maximum 25 mg for tenecteplase) are the recommended first-line intravenous thrombolytics in eligible patients with acute ischemic stroke treated within 4.5 h of symptom onset or last known well, whereas tenecteplase 0.4 mg/kg is not recommended. In contrast, reteplase currently has a limited role and may be considered only in selected patients not undergoing endovascular thrombectomy, based on still limited evidence [68].

### 2.4. The Safety of Antithrombotic Therapy

Bleeding complications represent a primary challenge in antithrombotic therapy. Strategies for their management include preventing potential bleeding episodes and, if they occur, therapeutic interventions involving general supportive treatment or the administration of specific neutralizing agents.

Efforts have been directed toward predicting bleeding risk by developing algorithms that incorporate pharmacogenetic data integrated with various blood-based parameters or patient clinical characteristics. Algorithms for warfarin dosage have been the most extensively investigated [20]. A systematic review of 433 such algorithms found that only 26% underwent external validation, and only 7% were evaluated for clinical utility. Furthermore, less than 2% were rated as having a low risk of bias [69]. Various bleeding risk assessment scales have also been developed for patients receiving anticoagulant therapy. Nevertheless, these scales often rely on complex scoring systems that pose challenges for practical clinical application. The HAS-BLED score was developed with the aim of user-friendliness and has subsequently become one of the most widely adopted and frequently utilized tools, particularly in patients with AF [70]. The VTE-BLEED score is increasingly relevant for patients with VTE and PE [71]. Other risk schemes, such as ATRIA [72] and those from the National Registry of Atrial Fibrillation (NRAF), have also been developed [73,74]. However, the capacity of these scales to accurately predict individual bleeding risk remains limited, and they should not serve as the sole determinant for therapeutic decision-making. The most well-known and commonly employed bleeding risk assessment scales in clinical practice are presented in Table 2.

The management of antithrombotic therapy can be guided by assessing clotting times or coagulation parameters. Monitoring is crucial for anticoagulants that are susceptible to interactions and fluctuations in therapeutic blood concentrations, such as warfarin and UFH. INR is used for warfarin dose adjustments, while the aPTT is used for UFH. Current guidelines generally advise against routine monitoring of anticoagulant activity for other antithrombotic agents [75,76]. However, measurement of anti-factor Xa activity for Xa inhibitors and thrombin time (TT), or preferably diluted TT, for thrombin inhibitors may be warranted in specific populations, including obese or pediatric patients, pregnant women, or those with renal impairment [77].

Several validated tools may help tailor antiplatelet therapy, particularly after PCI. PRECISE-DAPT, calculated at discharge or at the start of DAPT from age, creatinine clearance, hemoglobin, white-blood-cell count, and prior spontaneous bleeding, estimates bleeding risk; a score ≥ 25 identifies patients in whom a shorter DAPT strategy should be considered (Table 2). The DAPT score is applied after 12 months of event-free DAPT to estimate the net benefit of treatment extension beyond 1 year; scores ≥ 2 favor prolongation, whereas scores < 2 argue against it. In parallel, the ARC-HBR criteria provide a pragmatic binary definition of high bleeding risk (≥1 major or ≥2 minor criteria), corresponding to a ≥4% risk of BARC 3 or 5 bleeding or a ≥1% risk of intracranial hemorrhage at 1 year. The PARIS score may serve as an additional tool by separately estimating post-PCI bleeding and thrombotic risk. Importantly, these instruments should complement, not replace, individualized clinical assessment [78,79,80,81].

Monitoring antiplatelet drug activity is essential for assessing interindividual variability in response to therapy, particularly following PCI. Clinical evaluation often focuses on identifying high on-treatment platelet reactivity, which is strongly associated with an increased risk of ischemic events, such as stent thrombosis and myocardial infarction [82,83]. Common diagnostic methods include light transmission aggregometry, considered the gold standard, as well as point-of-care assays like multi-electrode aggregometry and VASP-based flow cytometry [84,85]. While routine monitoring is not generally recommended, it remains a valuable tool for risk stratification and personalized treatment adjustments in high-risk patients to balance ischemic protection and bleeding risks [86].

Despite efforts to mitigate bleeding risks associated with antithrombotic therapy, the incidence of minor bleeding events, such as conjunctival hemorrhages, epistaxis, gingival bleeding, and ecchymoses, can potentially exceed 10%. A meta-analysis of 27 RCTs involving 64,493 patients with VTE revealed that warfarin and rivaroxaban were associated with the highest risk of non-major bleeding, while apixaban demonstrated the lowest risk [87]. Evidence also suggests a higher risk of gastrointestinal bleeding in patients treated with rivaroxaban compared to apixaban [88]. However, the frequency of bleeding is multifactorial, extending beyond the pharmacological properties of the antithrombotic agent itself. The most severe bleeding complications include intracerebral hemorrhage and major gastrointestinal bleeding. Data from a large US healthcare system involving 69,000 patients indicate that major bleeding events with DOACs can range from several to 10% within the first five years of treatment in both inpatient and outpatient settings [89]. Landmark clinical trials in patients with AF, RE-LY, ENGAGE AF-TIMI 48, ROCKET AF, and ARISTOTLE reported a 1–2% risk of gastrointestinal bleeding within the first year of treatment [29]. Dabigatran has been associated with a lower incidence of life-threatening bleeds compared to warfarin; however, it exhibited a higher frequency of gastrointestinal bleeding [90]. The tartaric acid core released during dabigatran digestion may contribute to this, potentially causing mucosal injury by adhering to the esophageal lining, particularly in older, less active individuals with limited fluid intake [91]. Aspirin is also linked to a well-established risk of gastrointestinal bleeding, ranging from minor erosions to severe hemorrhages. This risk is further augmented by advanced age, the concomitant use of other antithrombotic or nonsteroidal anti-inflammatory drugs, a history of peptic ulcer disease, or a *Helicobacter pylori* infection. The underlying mechanism is attributed to the inhibition of protective gastric prostaglandins. Meta-analyses have demonstrated a 2- to 4-fold increased risk of upper gastrointestinal bleeding with aspirin use compared to non-use, with annual incidence rates ranging from 0.7% to 1.3%, depending on individual patient risk profiles [92]. Importantly, the clinical significance and severity of gastrointestinal bleeding are determined not only by the pharmacological profile of the antithrombotic agent but also by the bleeding site and extent, treatment duration, dosage, and individual patient characteristics.

Beyond bleeding complications, antithrombotic agents can also induce thrombocytopenia, defined as a platelet count below 150 × 10^9^/L. This condition may manifest asymptomatically or present with mucocutaneous bleeding, bruising, and petechiae, and in severe cases, can lead to life-threatening thrombosis [93]. Among drug-induced immune thrombocytopenias, heparin-induced thrombocytopenia (HIT) is a particularly significant clinical entity. HIT represents a distinct, immune-mediated disorder characterized by a paradoxical increase in thrombotic risk despite a reduction in platelet counts. Two recognized forms of HIT occur in heparin-treated patients: non-immune HIT (type 1), a transient and mild decrease in platelets without thrombotic sequelae, typically observed within the first five days of heparin exposure; and immune-mediated HIT (type 2), which usually develops between days 5 and 10 of therapy [94]. Type 2 HIT arises from IgG antibodies targeting platelet factor 4 (PF4)–heparin complexes, resulting in platelet activation via FcγRIIA receptors, the formation of procoagulant microparticles, and the generation of thrombin. Compared to patients without HIT, those with the condition face a heightened risk of thrombosis or bleeding, increased mortality, limb amputation, and prolonged hospitalization [95]. Beyond heparin, antiplatelet agents have also been implicated in inducing thrombocytopenia [96]. Given the potential for life-threatening complications, early recognition and immediate cessation of the implicated antithrombotic drug are critical in cases of suspected immune thrombocytopenia, followed by initiating an alternative anticoagulant [97]. Non-hemostatic adverse effects of antithrombotic agents may also occur, depending on the drug’s mechanism of action, route of administration, metabolic pathways, and interactions with other medications [98]. Beyond bleeding and non-bleeding adverse effects, all antithrombotic agents share a potential common outcome: a lack of therapeutic efficacy due to subtherapeutic plasma concentrations, which can arise from inappropriate dosing regimens, drug–drug interactions, or drug–food interactions. These diverse adverse effects underscore the critical importance of individualized treatment strategies, diligent monitoring, and dosage adjustments based on individual patient responses and clinical conditions.

### 2.5. Guideline-Based Optimization of Antithrombotic Regimens and Personalized Antiplatelet Therapy

Optimization of antithrombotic therapy is based less on isolated drug-versus-drug comparisons and more on guideline-directed selection of regimen intensity, duration, and combination according to the thrombotic setting and the patient’s net clinical profile. Current recommendations emphasize repeated reassessment of thrombotic or ischemic risk relative to bleeding risk, taking into account age, frailty, renal function, prior bleeding, active cancer, concomitant medications, and the presence of an indication for long-term oral anticoagulation [35,57,75,76,99,100,101].

In VTE, current guidance favors DOAC-based strategies for most patients without contraindications. When extended-phase anticoagulation is indicated, reduced-dose apixaban or rivaroxaban is preferred over aspirin or no treatment [100]. In cancer-associated VTE, both DOACs and LMWH remain acceptable options, but treatment should be individualized according to tumor site, gastrointestinal or genitourinary bleeding risk, oral absorption, drug–drug interactions, renal function, and the expected duration of active cancer [100] (Table 3).

Optimization of antiplatelet therapy is most evident after ACS and PCI. DAPT remains the default strategy, and prasugrel or ticagrelor is generally preferred over clopidogrel in ACS patients undergoing PCI when clinically suitable [35,57]. However, current guidelines also support personalization of DAPT duration and intensity. In patients who are event-free and not at high ischemic risk, transition to single antiplatelet therapy after 3–6 months can be considered, preferably with P2Y12 inhibitor monotherapy, whereas in patients at high bleeding risk, an even shorter DAPT course may be appropriate [35,57]. Conversely, prolonged intensified antithrombotic therapy may be considered in selected patients with high ischemic risk and acceptable bleeding risk [35,57,99,101].

In chronic coronary syndromes after elective PCI, the default strategy is shorter than in ACS, with aspirin plus clopidogrel for 6 months followed by single antiplatelet therapy, although 1–3 months may be sufficient in selected high-bleeding-risk patients [99]. In symptomatic peripheral artery disease or polyvascular atherosclerotic disease, low-dose rivaroxaban plus aspirin may be considered in patients without increased bleeding risk, particularly after lower-extremity revascularization [99,101].

Personalized antiplatelet therapy should therefore be explicitly recognized as a core element of modern management. In practice, it includes selection of the oral P2Y12 inhibitor according to clinical presentation and bleeding liability; tailoring DAPT duration to the individual balance between ischemic and hemorrhagic risk; de-escalating treatment intensity when bleeding concerns predominate; and, in selected ACS/PCI patients, considering CYP2C19-guided selection of oral P2Y12 inhibition when test results are expected to change management [35,57,102] (Table 3). The same individualized principle applies when AF coexists with coronary artery disease or recent PCI: current guidelines favor DOAC-based regimens, only a very short period of triple therapy, early transition to dual therapy with a single antiplatelet agent—preferably clopidogrel—and eventual oral anticoagulant monotherapy [75,76].

## 3. Future Strategies of Pharmacological Thrombosis Management

Targeting specific pathways to achieve a more potent antithrombotic effect and an improved safety profile, particularly for high-risk patients, aiming to mitigate bleeding risks and gastrointestinal toxicity, is desired. Although DOACs generally demonstrate comparable efficacy to warfarin with a reduced risk of major bleeding [103], studies comparing DOACs to LMWH in the context of cancer-associated VTE have revealed a trade-off. It has been previously proven that LMWHs were more effective than warfarin in reducing the risk of VTE recurrence in cancer patients without an increase in major bleeding [104,105,106]. The studies with edoxaban and rivaroxaban versus LMWHs suggest a potential reduction in recurrent VTE with DOACs but an increased risk of major and clinically relevant non-major bleeding events, frequently of gastrointestinal or urological origin [107,108]. Although Caravaggio’s study comparing apixaban with dalteparin showed similar effects without an increase in bleeding risk [109], the treatment of cancer-associated VTE remains a challenge [110].

Despite improvements in safety with the introduction of newer drugs, a subset of patients still cannot be adequately managed with currently available antithrombotic agents. Patients with mechanical heart valves are compelled to use older, more hazardous anticoagulants, such as warfarin, associated with significant inconveniences. The use of heparins in perioperative management, extracorporeal circulation, and hemodialysis also presents ongoing challenges. Novel technologies involving contact with artificial surfaces similarly necessitate anticoagulant protection. Current directions in enhancing the safety of anticoagulant therapy focus on discovering new drugs with a lower bleeding risk and developing strategies to restore normal hemostasis in the event of bleeding following anticoagulant administration.

### 3.1. Candidates for Novel Antithrombotic Agents

Anticoagulants currently in development are primarily antibodies targeting factors within the intrinsic coagulation pathway (Figure 2). This research aims to identify agents that, unlike existing anticoagulants, inhibit thrombosis development without affecting normal hemostasis. Inhibitors of FVIII or FIX were ineffective in clinical trials, leading to their early termination. The most promising candidates for next-generation anticoagulants remain inhibitors of factor XI. **ISIS416858**, an antisense oligonucleotide that inhibits factor XI synthesis, demonstrated significant antithrombotic activity and superiority over enoxaparin in patients undergoing orthopedic surgery or undergoing hemodialysis in a phase II trial [111]. Several monoclonal antibodies targeting factor XI are under investigation, such as **abelacimab**, which was evaluated in patients with AF in phase II studies and is currently in a phase III trial [112,113], and **osocimab**, an antibody binding to the catalytic domain of activated factor XI, which more effectively reduced the incidence of VTE compared to enoxaparin in patients undergoing total knee arthroplasty in a phase II trial [114]. Additionally, it is associated with a low risk of bleeding in patients with kidney failure undergoing hemodialysis [115].

However, a comprehensive assessment necessitates confirmation in larger studies. **Asundexian**, a small-molecule inhibitor of factor XI, reduced thrombosis in animal models [116]. When administered alone or in combination with an antiplatelet agent, it did not exacerbate bleeding, as confirmed in patients with a recent acute myocardial infarction [117], and it was associated with a lower risk of bleeding compared to apixaban in patients with AF [118]. Small-molecule pyridone-based FXIa inhibitors are also being preclinically investigated [119].

Thanks to a deeper understanding of the mechanisms underlying heparin’s anticoagulant activity, heparin-mimetic synthetic polymers incorporating sulfate, sulfonate, and carboxyl functional groups, with the potential to significantly impact thrombosis, have been evaluated in preclinical studies [120]. Our recent work in an animal model demonstrated that a block copolymer containing poly(sodium styrenesulfonate) (PSSS) and poly(ethylene glycol) (PEG) primarily targets factor XII and fibrinogen, suggesting potential applications in blood-contacting biomaterials for anticoagulation purposes [121]. Positively charged block copolymers can also exhibit anticoagulant activity [122]. Anionic polyP has been identified as a promising target for the development of novel cationic antithrombotic agents [123]. Follow-up research suggests that cationic polymers (MPI) may represent a compelling approach to developing new anticoagulants without bleeding effects, targeting polyP [124] (Figure 2). Despite these advancements, heparin’s broad applicability and reversibility, its well-established utility across a wide range of clinical indications, and its lower cost in many healthcare settings continue to underpin its fundamental role as an anticoagulant.

The novel candidates for antiplatelet agents are chemically diverse groups targeting well-established receptors, such as proteinase-activated receptor 1 (PAR-1), P2Y12, and GPIIb/IIIa, as well as emerging targets illustrated in Figure 1. Regarding PAR-1 receptor antagonists, **vorapaxar** was available for clinical use, but its safety profile was not sufficiently acceptable, limiting its adoption despite regulatory approval in 2014. Newer PAR-1 receptor antagonists, such as the pepducin **PZ-128**, and proteinase-activated receptor 4 (PAR-4) antagonists, such as **BMS-986120/BMS-986141**, exert a lesser impact on hemostasis and are currently in early clinical development [1]. A new candidate within GPIIb/IIIa receptor antagonists is **zalunfiban**, a small-molecule reversible platelet inhibitor administered subcutaneously. Its potential for self-administration and rapid onset of action suggests efficacy in the rapid pre-hospital treatment of myocardial infarction. It is currently in a phase III clinical trial [125]. Another candidate with a similar potential application is **selatogrel**, a selective, reversible P2Y12 receptor antagonist characterized by a rapid onset and short duration of action (Figure 1) [126]. Completing the ongoing phase III trial will indicate the efficacy and safety of self-administered subcutaneous selatogrel in patients with suspected myocardial reinfarction. New targets of interest include GPI and GPVI. **Anfibatide**, a protein derived from snake venom, competitively binds to GPIbα and blocks vWF binding. Preclinical studies in murine models suggested its potential to inhibit thrombosis without increasing bleeding. A completed phase II study in patients with non-ST-segment elevation myocardial infarction undergoing PCI confirmed its safety [127]. Other molecules, such as **caplacizumab**, which affects the GPIb–GPV–GPIX–vWF axis and is registered for treating episodes of acquired thrombotic thrombocytopenic purpura, or anti-P-selectin monoclonal antibodies targeting blood cell adhesion, are also in development [46,128]. GPVI appears to be a promising target for antiplatelet therapy, potentially reducing atherothrombosis with minimal impact on hemostasis. **Revacept**, a dimeric soluble fusion protein containing the extracellular domain of GPVI, competes with endogenous GPVI for binding to collagen and vWF, thereby interfering with the platelet response to exposed collagen (Figure 1). A Phase II clinical trial conducted in patients undergoing elective PCI for stable ischemic heart disease showed reduced bleeding, with no significant differences between treatment arms reported regarding efficacy [129]. **Glenzocimab**, a humanized monoclonal antibody that inhibits GPVI, exhibited antithrombotic effects similar to those of aspirin and P2Y12 inhibitors but with a lower impact on bleeding [39]. Recent research has indicated that glenzocimab may be effective and safe in combination with alteplase in patients with acute ischemic stroke [130]. It is also worth mentioning **VLX-1005** (previously known as ML355), a novel 12-lipoxygenase (12-LOX) inhibitor designed to prevent immune-mediated platelet activation and the development of HIT [131], which is currently in a Phase II study planned for patients with suspected HIT. A Phase II trial demonstrated that isoquercetin, an inhibitor of protein disulfide isomerase, could protect patients with advanced cancer from developing VTE [132]. Emerging promising targets and active compounds binding to them include intracellular kinases such as phosphoinositide 3-kinase-β (PI3Kβ), tyrosine-protein BTK kinase, and casein kinase [46]. Drug candidates presented in Figure 1 are currently in the research phase, and whether they will translate into effective antiplatelet agents without significant bleeding risks necessitates further investigation.

Novel thrombolytics are being developed to replace alteplase, including mutation variants of alteplase (lanoteplase, monteplase, duteplase, YM866, and amideplase) or non-alteplase derivatives (staphylokinase and desmoteplase) [61]. The aim is to develop an agent with a longer half-life, improved fibrin specificity, a faster onset of action, and greater ease of administration as a single or double bolus.

Among agents in development, **lanoteplase** has been the most extensively studied thus far. Possessing a long plasma half-life of up to 45 min, it could be administered as a single bolus. However, the InTIME-2 trial reported an increased rate of hemorrhagic stroke [133]. Like many other mutation variants, the lack of demonstrated superiority over alteplase has hindered its development. Other innovative strategies in development include advanced drug delivery systems (e.g., pegylation, dendrimers, or liposomes), combination therapy with antiplatelet agents, and adjunct therapies aimed at mitigating the neurotoxicity of t-PA [61].

A different target of novel thrombolytics is activated thrombin-activatable fibrinolysis inhibitor (TAFI). TAFI, activated by the thrombin–thrombomodulin complex, attenuates fibrinolysis by removing C-terminal lysine and arginine residues from partially degraded fibrin. These residues augment fibrinolysis by providing additional binding sites for plasminogen and t-PA. Inhibition of TAFI prevents this removal, thereby enhancing fibrinolysis. **DS-1040**, a small-molecule inhibitor of TAFI, was well-tolerated in a Phase I study involving both young and elderly subjects, with no observed increase in bleeding time (Figure 2) [134]. A second target for enhancing endogenous fibrinolysis is α2-antiplasmin, which inhibits plasmin; therefore, inhibiting α2-antiplasmin may promote clot lysis. Monoclonal antibodies against α2-antiplasmin have shown promise in animal models. **TS-23 (DS-9231)**, an α2-antiplasmin-inactivating antibody, was well-tolerated in a first-in-human study, suppressing α2-antiplasmin activity without bleeding complications (Figure 2) [110]. A phase II trial is ongoing to evaluate safety and the thrombolytic effect in subjects with intermediate-risk (sub-massive) acute PE. Approved thrombolytic agents and those in development are presented in Figure 2.

### 3.2. Neutralization of Antithrombotic Effect as an Alternative Approach to Improve the Safety of Therapy

Life-threatening bleeding in patients receiving antithrombotic agents may require fast neutralization of the anticoagulant activity. The clinical decision to antagonize the antithrombotic effect hinges on several factors, including the anticipated bleeding risk associated with a procedure, the anatomical site of the hemorrhage, and the urgency of any required surgical intervention. Traditional, non-specific approaches involve the transfusion of blood products. While coagulation factor concentrates effectively restore coagulation following the administration of warfarin, urgent reversal is not always required. A particularly critical patient subgroup includes those experiencing anticoagulant-associated intracerebral hemorrhage, a condition characterized by increased hematoma volumes, a heightened risk of secondary hematoma expansion, and elevated morbidity and mortality. In this case, the primary reversal strategy involves administering vitamin K in conjunction with prothrombin complex concentrates (PCCs). Although fresh frozen plasma (FFP) or recombinant activated factor VIIa (rFVIIa) have been explored for this purpose, PCCs are preferred over FFP [135], and current guidelines do not recommend the use of rFVIIa [136].

**Figure 3:**
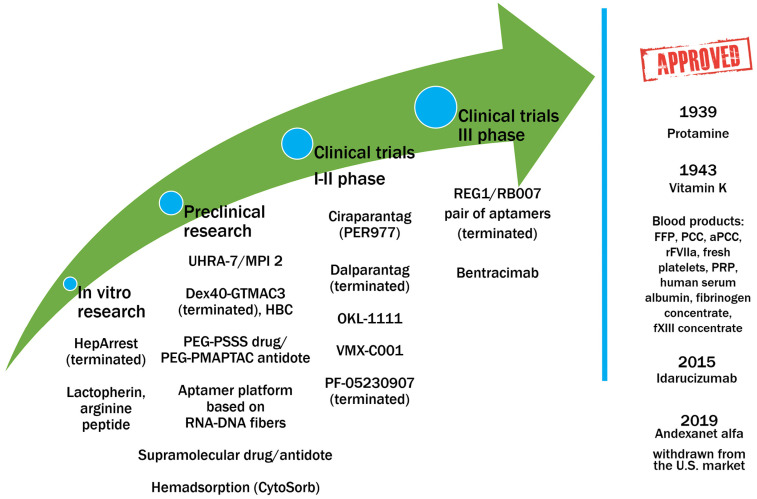
The development pipeline of antithrombotic reversal agents. UHRA-7—Universal Heparin Reversal Agent-7; MPI 2—macromolecular polymer inhibitor-2; Dex40-GTMAC3—40 kDa dextran substituted with glycidyltrimethylammonium chloride-3; HBC—Heparin Binding Copolymer; PEG—poly(ethylene glycol); PMAPTAC—poly((3-(methacryloylamino)propyl)trimethylammonium chloride); PSSS—poly(sodium styrenesulfonate); OKL-1111—octakis-(6-((6-carboxyhexyl)thio)-6-deoxy)-gamma-cyclodextrin; FFP—fresh frozen plasma; PCC—prothrombin complex concentrates; rFVIIa—recombinant activated factor VIIa; aPCC—activated PCC; PRP—platelet-rich plasma.

Until recently, protamine was the primary specific agent used to reverse bleeding after anticoagulant administration. However, its efficacy is mainly limited to restoring hemostasis following UFH administration. Its neutralizing effect is significantly reduced, by almost half, in the case of LMWHs. Heparins, particularly UFH, are utilized in emergency and inpatient settings requiring strong anticoagulation, such as during vascular surgery or extracorporeal circulation. In these situations, as well as in cases of heparin overdose, intracerebral hemorrhage, or trauma, protamine administration may be necessary. Around 4 million packages of protamine are used annually, indicating its continued clinical relevance. Beyond its limited efficacy against LMWHs and fondaparinux, the use of protamine is further constrained by its cardiorespiratory and immunological toxicity, linked to its chemical structure and origin [137]. We have demonstrated direct cardiotoxicity of protamine in zebrafish and rodents [138]. Similar effects have been reported in patients, although these are primarily case reports [139]. Nonetheless, protamine administration still carries risks associated with determining the appropriate dose to effectively reverse UFH. Exceeding a 1:1 ratio can paradoxically lead to heparin-like anticoagulant effects. The antithrombotic potential of protamine may contribute to an increased bleeding risk [140]. These findings underscore the clinical need for a protamine alternative to neutralize UFH, LMWHs, and fondaparinux. Polysaccharide-based inhibitors and cationic block copolymers, such as heparin-binding copolymer (HBC), demonstrated potential as broad-spectrum heparin antidotes [141,142,143,144]. Pursuing an effective and universal heparin reversal agent remains an active area of investigation. **Ciraparantag**, a cationic synthetic molecule, was designed as an antidote for heparins but also exhibits inhibitory activity against apixaban and rivaroxaban [145,146]. **UHRA (Universal Heparin Reversal Agent)** and synthetic dendritic polymers that reverse the anticoagulant activity of all available heparins are in the preclinical research phase [147,148] (Figure 3).

The increasing adoption of DOACs spurred manufacturers to develop specific reversal agents to enhance their safety [149]. The availability of these antidotes also likely contributed to the broader acceptance of DOACs in clinical practice. **Idarucizumab**, a monoclonal antibody Fab fragment that binds specifically to dabigatran, received FDA approval in 2015 for reversal of dabigatran’s effects in patients with life-threatening bleeding or requiring urgent surgery [150]. Subsequently, **andexanet alfa**, a recombinant fXa variant lacking enzymatic activity, was approved in 2019 based on the ANNEXA trials. Andexanet alfa effectively neutralizes the activity of fXa inhibitors, including apixaban, rivaroxaban, and enoxaparin [150,151]. However, a recent study indicated that andexanet alfa treatment was associated with an increased incidence of thromboembolic events, a finding that may be influenced by the study’s open-label design [152]. Notably, in December 2025, andexanet alfa was withdrawn from the U.S. market after the FDA concluded that the overall risk-benefit profile was unfavorable, mainly due to postmarketing and trial data showing an increased risk of thromboembolic events [153]. Thus, despite these advancements, the optimal management of DOAC-related bleeding remains a subject of ongoing investigation. The superiority of specific reversal over non-specific hemostatic treatments in emergencies remains unclear due to a lack of comparative, prospective, randomized studies [154].

Specific reversal agents for Xa inhibitors under development, beyond ciraparantag and andexanet alfa, include variants of factor Xa. Among them is **VMX-C001**, a recombinant factor Xa analog inspired by the *Pseudonaja textilis* snake venom sequence, which has demonstrated efficacy and safety in preclinical studies. A Phase I clinical trial for VMX-C001 was completed in 2023; however, the results remain unpublished [155]. Another activated factor Xa variant, **PF-05230907**, was evaluated in a phase 1b trial involving patients with intracerebral hemorrhage; however, this study was prematurely terminated due to challenges in recruiting patients with ongoing intracerebral hemorrhage [156].

More recent research focuses on different approaches to reverse the effects of anticoagulant drugs. A novel anticoagulant platform based on aptamers and specific neutralizers has been developed, and its advantages in dialysis and during cardiac and other surgical procedures were presented in animal models [157]. Another research group demonstrated the utility of combinatorial chemistry in screening ligands that bind to heparin [158]. Furthermore, by selectively choosing the identity and modulating the density of cationic binding groups on the polymer scaffold, a superior universal heparin reversal agent (MPI 2) with improved heparin-binding activity and enhanced hemocompatibility profiles, leading to a minimal effect on hemostasis, has been proposed [159]. Notably, advanced research with a modified cyclodextrin—OKL-1111—demonstrates the potential to neutralize the effects of both anticoagulants and antiplatelet drugs and is already in the clinical phase [160] (Figure 3).

Several groups have been working on new anticoagulants alongside their compatible antidote. One such endeavor was pegnivacogin (REG1), an inhibitory RNA aptamer directed against FIXa, which could be rapidly neutralized by a complementary aptamer, anivamersen (RB007). Phase I and II studies with REG1 showed promise. Still, the phase III REGULATE PCI trial was prematurely terminated after partial enrollment due to severe allergic reactions, which halted further development of REG1 [161]. A similar strategy to achieve on-demand reversibility involved designing a supramolecular drug whose activity could be reversed by an antidote that competitively inhibits the hybridization between its fragments. This supramolecular inhibitor effectively suppressed thrombus formation in a murine needle injury thrombosis model, and its activity was subsequently reversed [162]. In parallel, we explored the development of a complementary anticoagulant-antidote system using block copolymers. The anticoagulant was composed of a neutral PEG block and various anionic blocks based on PAMPS (poly(sodium 2-acrylamido-2-methylpropanesulfonate)); the antidote was composed of PEG and a cationic block based on PMAPTAC (poly((3-(methacryloylamino)propyl)trimethylammonium chloride)) [163]. Our recent research yielded an anticoagulant copolymer based on PEG and poly(sodium styrenesulfonate) (PSSS) blocks with enhanced efficacy, as confirmed in rodent models following intravenous and subcutaneous administration. Notably, we also demonstrated that the anticoagulant activity of an anionic copolymer was completely reversed by a cationic copolymer, PEG-PMAPTAC, in both rat and human plasma [121].

The occurrence of major bleeding into critical organs in patients on antiplatelet therapy presents a significant clinical challenge, particularly during trauma or unscheduled invasive procedures. In scenarios requiring emergency surgery, the increased risk of bleeding against the potential for ischemic events must be weighed if antiplatelet therapy is discontinued. Platelet transfusion is a general recommendation for life-threatening bleeding [164,165], demonstrating a more pronounced effect in patients treated with clopidogrel or prasugrel compared to ticagrelor. Unlike other P2Y12 inhibitors, ticagrelor’s reversible binding to platelets raises concerns that residual drug would inhibit transfused platelets, limiting their efficacy. Thus, developing specific reversal agents for ticagrelor is a promising avenue [166,167].

**Bentracimab** (PB2452), a monoclonal antibody fragment with high affinity for ticagrelor and its active metabolite, demonstrated complete and sustained reversal of ticagrelor’s antiplatelet effects within minutes in a phase I trial with healthy volunteers without evidence of rebound platelet activity and with limited adverse events [166,168]. The REVERSE-IT trial further confirmed the immediate and sustained reversal of ticagrelor’s antiplatelet effects with bentracimab in patients requiring urgent surgery or experiencing major hemorrhage with no reported allergic or infusion-related reactions (Figure 3) [169]. Alternative strategies for ticagrelor reversal include hemostatic agents (recombinant activated factor VII, fibrinogen concentrate, tranexamic acid, factor XIII concentrate) [170,171] and sorbent hemadsorption [170,172,173]. In vitro studies have shown that standard hemostatic agents do not affect platelet aggregation in blood treated with ticagrelor. However, sorbent hemadsorption with CytoSorb demonstrated efficient in vitro removal of ticagrelor from human blood. Case series in patients undergoing urgent coronary artery bypass grafting with CytoSorb integration within the cardiopulmonary bypass circuit have been described. Still, prospective studies are needed to confirm the safety and efficacy of this approach.

## 4. Conclusions

We currently have a range of antithrombotic agents available that prevent or treat patients with thromboembolic disorders. Despite advancements made after the year 2000, a significant portion of patients still require gold-standard drugs such as heparin, warfarin, or aspirin. For these individuals, therapy presents considerable inconveniences, including the necessity of injections, regular diagnostic testing, attention to drug interactions and diet, as well as bleeding from the gums, eyes, or gastrointestinal tract. In some patients receiving heparins, life-threatening adverse effects like intracerebral bleeding or thrombocytopenia may occur, necessitating drug discontinuation and alternative newer therapies. In contrast to the constant INR monitoring required with warfarin, newer drugs like DOACs do not necessitate continuous monitoring. DOACs are also less prone to interactions with other medications, food, and genetic variability in patients. However, a subset of patients taking these newer agents remains at risk of major bleeding, which is associated with a several-fold increase in ischemic events and death, reflecting, in part, the interruption of antithrombotic therapy [174]. In such cases, neutralizing the anticoagulant effect and resuming therapy after the threat has subsided may be necessary.

Current research directions address these needs by seeking new drugs with maximally reduced bleeding risks or other adverse effects. However, a key challenge in introducing novel antithrombotic agents lies in demonstrating superior or, at the very least, equivalent efficacy with enhanced safety compared to existing antiplatelet and anticoagulant medications. The substantial financial investment and logistical complexities of large-scale clinical trials further complicate this endeavor. Researchers are investigating specific clinical niches where novel agents can be evaluated against placebo or problematic standard-of-care treatments to overcome these obstacles. Examples include exploring fXI/XII inhibitors for central venous catheter thrombosis prophylaxis in cancer patients and VTE prevention in patients with active malignancy or undergoing hemodialysis. The judicious selection of well-defined patient populations and the use of innovative trial designs are crucial for the regulatory approval of next-generation antithrombotic agents. Furthermore, a comprehensive understanding of the diverse triggers and mechanisms of thrombogenesis across various patient populations and anatomical locations is essential for developing targeted and more efficacious strategies. The development of new antiplatelet drugs like subcutaneous zalunfiban and selatogrel for rapid pre-hospital treatment of ST-segment elevation myocardial infarction also exemplifies this trend. Following the registration of idarucizumab and andexanet alfa, the search for new specific anticoagulant reversal agents continues, exemplified by bentracimab, nearing FDA approval for neutralizing ticagrelor’s antiplatelet activity.

It appears that the latest research directions will focus on filling gaps in available antithrombotic therapy for specific patient groups, such as those with cancer or end-stage renal disease, pregnant women, patients undergoing hemodialysis, or cardiovascular surgery. The unresolved issues surrounding implanted devices, like artificial heart valves, suggest a future direction for novel solutions.

## Figures and Tables

**Table 1:** Currently available antithrombotic agents and their basic characteristics.

Name	Molecular Weight (kDa)	Chemical Nature	Prodrug	Mean Half-Life After a Single Dose
		**Parenteral**		
		**Anticoagulants**		
unfractionated heparin	mean of about 12–14 (ranging from 2 to 40)	polysaccharide	No	1.5 h
enoxaparin, dalteparin, nadroparin	3.6—6.5	polysaccharide	No	5 h
				7 h
				7 h
fondaparinux	1.7	pentasaccharide	No	19 h
bivalirudin	2.2	oligopeptide	No	25 min
argatroban	0.5	heterocyclic derivative of L-arginine	No	40 min
		**Antiplatelet agents**		
cangrelor	0.77	nucleoside triphosphate analog	No	4.2 min
eptifibatide	0.8	cyclic heptapeptide	No	2.5 h
tirofiban	0.5	heterocyclic derivative of L-tyrosine	No	2 h
		**Thrombolytic agents**		
alteplase	67–70	glycoprotein	No	3.5 min
reteplase	39	nonglycosylated peptide	No	12.6 min
tenecteplase	65	glycoprotein	No	22 min
		**Oral**		
		**Anticoagulants**		
warfarin	0.3	coumarin derivative	No	36 h
dabigatran	0.47	aromatic amide/benzimidazole derivative	Yes	8 h
rivaroxaban	0.44	aromatic amide/thiophene derivative	No	7 h
apixaban	0.46	aromatic amide/pyrazolopyridine derivative	No	12 h
edoxaban	0.55	oxamide/chloropyridine/ thiazolopyridine	No	12 h
		**Antiplatelet agents**		
aspirin	0.18	acetyl derivative of salicylic acid	No	20 min
clopidogrel	0.42	thienopyridine derivative	Yes	6 h
prasugrel	0.41	thienopyridine derivative	Yes	7 h
ticagrelor	0.52	cyclopentyl-triazolopyrimidine	No	6.3 h

h—hours; min—minutes.

**Table 2:** Bleeding Scales.

Scale Name	Indication	Purpose	Key Components
HAS-BLED	AF	Annual risk of major bleeding	Hypertension, Abnormal renal/liver function, Stroke, History of or predisposition to bleeding, Labile INRs, Elderly (>65), Drugs/alcohol
ORBIT	AF	Major bleeding risk	Age, ↓ Hb/Hct/Anemia hx, Bleeding hx, Impaired renal function. Antiplatelet therapy
ATRIA	AF	Bleeding risk	Anemia, Severe renal impairment, Age ≥ 75, Previous bleeding, Hypertension
HEMORR_2_HAGES	AF	Bleeding risk	More complex: Genetic factors, ↓ Hct/Anemia, Older age, Alcohol abuse, etc.
VTE-BLEED	VTE, PE	Risk of major bleeding during VTE	Active cancer, Age ≥ 60, Male, Anemia, Bleeding hx, Renal impairment, Antiplatelet therapy
PRECISE-DAPT	Patients treated with DAPT after PCI	Bleeding risk stratification and support for DAPT duration individualization	Age, creatinine clearance, Hb, white-blood-cell count, prior spontaneous bleeding

AF—Atrial fibrillation; VTE—Venous Thromboembolism; PE—Pulmonary Thromboembolism; INRs—International Normalized Ratio; Hb—Hemoglobin; Hct—Hematocrit; DAPT—dual antiplatelet therapy; PCI—percutaneous coronary intervention; ↓—decrease.

**Table 3:** Guideline-Based Optimization of Antithrombotic Therapy and Personalized Antiplatelet Therapy across Major Thrombotic Settings.

Clinical Setting	Default Guideline-Based Strategy	Optimization/Personalization for Clinical Translation	Key Refs.
Extended secondary prevention after VTE	Extended anticoagulation with reduced-dose apixaban or rivaroxaban when indicated	Aspirin should not replace indicated anticoagulation; full-dose extended therapy should be reserved for selected patients with very high recurrence risk and acceptable bleeding risk	[100]
Cancer-associated VTE	DOAC or LMWH	Individualize according to tumor site, gastrointestinal/genitourinary bleeding risk, oral absorption, drug–drug interactions, renal function, patient preference, and persistence of active cancer	[100]
ACS after PCI, without chronic OAC indication	12 months of DAPT; prasugrel or ticagrelor preferred over clopidogrel when suitable	Shorten DAPT in HBR; consider SAPT or P2Y12 inhibitor monotherapy after 3–6 months in event-free patients without high ischemic risk; prolong therapy in selected high-ischemic-risk patients without HBR	[35,57]
CCS after elective PCI	Aspirin + clopidogrel for 6 months, then SAPT	1–3 months of DAPT may be sufficient after non-complex PCI or in selected HBR patients	[99]
High-risk CCS/polyvascular atherosclerotic disease without HBR	Long-term SAPT	Consider dual-pathway inhibition with aspirin + rivaroxaban 2.5 mg twice daily in selected high-thrombotic-risk patients	[99,101]
Symptomatic PAD/after lower-extremity revascularization	SAPT with aspirin or clopidogrel	Consider low-dose rivaroxaban + aspirin in patients without increased bleeding risk; after endovascular revascularization, DAPT for 1–6 months may be used	[101]
AF without recent ACS/PCI	OAC monotherapy; DOAC preferred in most patients	Antiplatelet therapy should not be used instead of indicated OAC for stroke prevention	[75,76]
AF + recent ACS/PCI	Very short triple therapy, then DOAC + single antiplatelet therapy, then OAC alone	Prefer clopidogrel as the antiplatelet partner; minimize triple-therapy duration according to bleeding risk and stent-thrombosis risk	[75,76]
Selected ACS/PCI patients eligible for precision P2Y12 therapy	Standard clinical selection of the oral P2Y12 inhibitor	Consider CYP2C19-guided P2Y12 selection or de-escalation when test results are expected to change management	[35,102]

ACS—acute coronary syndrome; AF—atrial fibrillation; CCS—chronic coronary syndrome; DAPT—dual antiplatelet therapy; DOAC—direct oral anticoagulant; HBR—high bleeding risk; LMWH—low-molecular-weight heparin; OAC—oral anticoagulant; PAD—peripheral artery disease; PCI—percutaneous coronary intervention; SAPT—single antiplatelet therapy; VTE—venous thromboembolism.

## Data Availability

No new data were created or analyzed in this study.

## Abbreviations

The following abbreviations are used in this manuscript:

TF	Tissue factor
vWF	von Willebrand factor
t-PA	Tissue plasminogen activator
PAI-1	Plasminogen activator inhibitor-1
polyP	Polyphosphates
NET	Neutrophil extracellular traps
PE	Pulmonary embolism
VTE	Venous thromboembolism
HIT	Heparin-induced thrombocytopenia
VKORC1	Epoxide reductase complex subunit 1
INR	International normalized ratio
PT	Prothrombin time
DOACs	Direct oral anticoagulants
ACS	Acute coronary syndrome
DAPT	Dual antiplatelet therapy
PCI	Percutaneous coronary intervention
aPTT	Activated partial thromboplastin time
TT	Thrombin time
PF4	Platelet factor 4
PAR-1	Proteinase-activated receptor 1
PAR-4	Proteinase-activated receptor 4
12-LOX	12-lipoxygenase
